# Association of Maintenance Therapy Strategies with Survival Following First-Line Bevacizumab-Based Combination Chemotherapy in Metastatic Colorectal Cancer: A Single-Center Retrospective Observational Study

**DOI:** 10.3390/jcm15166279

**Published:** 2026-08-13

**Authors:** Simay Çokgezer, Burak Şakar, Senem Karabulut

**Affiliations:** Department of Medical Oncology, Institute of Oncology, Istanbul University, 34093 Istanbul, Turkey; buraksakar@gmail.com (B.Ş.); drsenemkarabulut@gmail.com (S.K.)

**Keywords:** bevacizumab, real-world data, maintenance therapy, metastatic colorectal cancer

## Abstract

**Objectives:** This study evaluated the association between maintenance therapy and survival outcomes following first-line bevacizumab-based combination chemotherapy in metastatic colorectal cancer (mCRC) using real-world data. **Methods:** This retrospective study included 94 mCRC patients achieving disease control, defined as complete response (CR), partial response (PR), or stable disease (SD), after first-line bevacizumab-based chemotherapy (2015–2025). Patients were divided into maintenance (fluoropyrimidine ± bevacizumab, n = 44) and active surveillance (n = 50) groups. Progression-free survival (PFS) and overall survival (OS) were analyzed using a landmark approach, Kaplan–Meier methods, and Cox regression models. **Results:** The overall patient cohort (n = 94) had a median age of 62 years and a male predominance of 61.7%. Median OS was significantly longer with maintenance therapy than surveillance (24.8 vs. 18.0 months; *p* = 0.045); PFS differences were not statistically significant (11.3 vs. 6.7 months; *p* = 0.077). In multivariable analysis, maintenance was not independently associated with OS or PFS. Male sex, comorbidities, multiple metastases, and non-resected primary tumors independently predicted worse OS. No statistically significant treatment-by-subgroup interactions were identified in the exploratory analyses. **Conclusions:** These real-world data suggest a potential survival advantage associated with maintenance therapy following first-line bevacizumab-based combination chemotherapy in patients with mCRC who achieve disease control. However, this association was not maintained after multivariable adjustment, suggesting that baseline differences may have contributed to the observed findings. Trial Registration: This retrospective observational study was not prospectively registered.

## 1. Introduction

Colorectal cancer (CRC) is the third most commonly diagnosed malignancy worldwide and the second leading cause of cancer-related mortality [1]. Approximately 1.1–2 million new cases are reported annually, with 15–30% of patients presenting with metastatic disease at diagnosis (synchronous metastases), while 20–50% of those initially diagnosed with localized disease develop metastases during the course of their illness [1,2]. In recent years, advances in systemic therapies and the integration of biological agents into chemotherapy regimens have led to a significant transformation in the management of metastatic colorectal cancer (mCRC), resulting in meaningful improvements in disease control and survival outcomes [3].

Currently, first-line treatment of mCRC consists of chemotherapy doublets combining a fluoropyrimidine with either oxaliplatin or irinotecan (FOLFOX, FOLFIRI, or CAPOX/XELOX), administered in combination with targeted agents in appropriately selected patients [4]. In this context, the addition of anti-vascular endothelial growth factor (anti-VEGF) agents such as bevacizumab enhances treatment efficacy by inhibiting angiogenesis and provides clinically meaningful benefits in both progression-free survival (PFS) and overall survival (OS) [5].

The “induction and maintenance” strategy in mCRC management has emerged in response to the need to limit cumulative toxicities associated with prolonged full-dose chemotherapy [1,4,6,7]. Maintenance therapy aims to sustain the response or disease stabilization achieved with induction therapy while continuing treatment with reduced toxicity and preserving quality of life (QoL) [4]. Historically, the standard approach involved continuous full-dose treatment until disease progression or unacceptable toxicity; however, this strategy has been associated with increased adverse effects and potential development of resistance [4,8]. Conversely, complete discontinuation of therapy after induction may lead to loss of disease control. Therefore, current strategies increasingly focus on maintenance approaches that aim to preserve disease control using lower-intensity treatment regimens [3].

Existing clinical evidence suggests that bevacizumab-based maintenance therapy provides a clear benefit, particularly in terms of PFS in mCRC [9,10]. However, its impact on OS appears to be more limited and often fails to reach statistical significance [4]. Phase III trials such as CAIRO3 and AIO KRK 0207 further support these findings [11,12]. Accordingly, while current data support the efficacy of maintenance therapy, they also highlight the need to individualize optimal treatment strategies based on patient characteristics and treatment tolerability.

Although randomized phase III trials form the basis of treatment guidelines in mCRC, their generalizability to real-world clinical practice is limited due to the selection of patient populations. Clinical trials typically include younger patients with fewer comorbidities, whereas patients encountered in routine practice tend to be older and more heterogeneous. Therefore, real-world evidence (RWE) plays a crucial complementary role in evaluating the effectiveness of maintenance therapy and understanding treatment selection under routine clinical conditions in heterogeneous patient populations that are underrepresented in randomized clinical trials [13].

In this retrospective real-world observational study, we aimed to evaluate the association between maintenance therapy and survival outcomes following first-line bevacizumab-based combination chemotherapy in patients with mCRC. Specifically, survival outcomes of patients receiving maintenance therapy were compared with those of patients managed with active surveillance to evaluate the effectiveness of maintenance therapy and describe treatment selection in routine clinical practice. Unlike randomized clinical trials conducted in highly selected populations, this study reflects routine clinical practice by evaluating a heterogeneous real-world cohort of patients achieving disease control after first-line induction therapy. Given its observational design, this study should be considered hypothesis-generating rather than confirmatory. We hypothesized that maintenance therapy would be associated with survival outcomes after adjustment for clinically relevant prognostic factors in a heterogeneous real-world population.

## 2. Materials and Methods

### 2.1. Study Design and Patient Selection

This study was designed as a single-center, retrospective, observational analysis to evaluate the association between maintenance therapy and survival outcomes in patients with mCRC following first-line bevacizumab-based combination chemotherapy.

Patients aged ≥18 years with histopathologically confirmed metastatic colorectal adenocarcinoma diagnosed between January 2015 and December 2025 and followed at the Department of Medical Oncology, Institute of Oncology, Istanbul University, were included in the study. Clinical, pathological, treatment, and follow-up data were retrospectively extracted from the institutional electronic medical records between March and May 2026. Complete baseline clinicopathological characteristics, treatment information, radiological response assessment, and follow-up data were required for study inclusion. The data cutoff date for all analyses was 31 December 2025. Eligible patients were those who received first-line combination chemotherapy with bevacizumab and achieved disease control, defined as complete response (CR), partial response (PR), or stable disease (SD), upon treatment evaluation. Since maintenance therapy is a clinically applicable strategy only in patients achieving disease control, those with primary progressive disease were excluded.

A total of 94 patients with complete clinical, pathological, and follow-up data were included and categorized into two groups: those who received maintenance therapy (n = 44) and those who underwent active surveillance (n = 50). All patients meeting the eligibility criteria during the study period were included; no sampling procedure was applied.

### 2.2. Assessment of Clinical and Treatment Characteristics

Demographic and clinical data were retrospectively obtained from the institutional electronic medical records. The variables evaluated included age at diagnosis, sex, performance status, tumor location, metastatic disease burden and distribution, surgical status of the primary tumor and metastatic sites, administered chemotherapy regimens, use of anti-VEGF therapy, and maintenance treatment status. Comorbidity was classified as present when at least one chronic coexisting medical condition was documented in the medical history, including hypertension, diabetes mellitus, coronary artery disease, cardiac arrhythmia, inflammatory bowel disease, or other chronic cardiovascular, respiratory, renal, hepatic, endocrine, neurologic, rheumatologic, or urologic conditions. Comorbidity status was recorded as a binary variable (present/absent); a validated comorbidity index such as the Charlson Comorbidity Index could not be retrospectively reconstructed.

In addition, molecular characteristics of the tumor were assessed, including RAS (KRAS/NRAS) and BRAF mutation status, as well as microsatellite instability (MSI) status. Molecular results were obtained from the available pathology or molecular diagnostic reports in the medical records, including reports from outside institutions when available. Testing was generally performed at diagnosis and repeated when clinically indicated. Because this retrospective study included patients diagnosed over a 10-year period, detailed information regarding the testing platform was not consistently available. Because BRAF and MSI data were unavailable for a substantial proportion of patients, these variables were not included in the statistical analyses. RAS status was available for 41 of 44 patients in the maintenance group and 42 of 50 patients in the active surveillance group, corresponding to 3 and 8 patients with missing RAS data, respectively. Exact tested and missing denominators for BRAF and MSI could not be reliably reconstructed retrospectively because these tests were not systematically recorded in the study database, particularly during the earlier years of the study period. The principal reason for missing BRAF and MSI information was that these tests were not routinely performed during the earlier years of the study period; patient-specific reasons for missing testing were not consistently documented.

All patients received first-line fluoropyrimidine-based combination chemotherapy (FOLFOX, FOLFIRI, CAPOX, CAPIRI, or FOLFOXIRI) in combination with bevacizumab. Post-first-line treatment strategies were determined based on the patient’s clinical condition, treatment tolerability, and physician and patient preference.

In patients receiving maintenance therapy, the regimens included capecitabine, capecitabine plus bevacizumab, and 5-fluorouracil/folinic acid (5-FU/FA) plus bevacizumab. At our institution, maintenance therapy was generally offered to patients who achieved disease control following first-line bevacizumab combination chemotherapy, had no unacceptable treatment-related toxicity precluding continuation of fluoropyrimidine-based therapy, and agreed to continue treatment after discussion with the treating physician. Maintenance therapy was generally not pursued in patients who declined further treatment or developed treatment-related toxicities that precluded continuation of maintenance therapy.

Because of the relatively limited number of patients within each maintenance regimen, the primary analysis was designed to evaluate the overall strategy of fluoropyrimidine-based maintenance therapy with or without bevacizumab versus active surveillance, rather than to compare individual maintenance regimens. Although these regimens differ biologically and clinically, they share the common therapeutic objective of maintaining disease control after induction therapy while reducing treatment intensity. Therefore, they were analysed as a single maintenance cohort in the primary analysis, whereas regimen-specific comparisons were considered exploratory.

Patients managed with active surveillance underwent outpatient follow-up every three months, including clinical evaluation, laboratory investigations (including tumor marker assessment), and contrast-enhanced computed tomography. No systemic anticancer treatment was administered during the surveillance period unless disease progression was documented, at which point subsequent-line treatment was initiated. Patients receiving maintenance therapy underwent routine clinical and laboratory assessments before each treatment cycle, while radiological follow-up was performed approximately every three months, according to the same institutional schedule as the active surveillance group.

### 2.3. Follow-Up and Endpoints

Patients were followed with regular clinical and radiological assessments. To minimize immortal time bias, a landmark analysis approach was employed. The landmark time was defined as the date of the first radiological response evaluation following completion of first-line treatment, representing the clinical decision point for maintenance therapy. Response assessment was routinely performed approximately two weeks after completion of first-line bevacizumab-based combination chemotherapy. This assessment served as the landmark time point for both treatment groups and represented the clinical decision point for maintenance therapy or active surveillance.

Patients who were alive, progression-free, and had achieved disease control (CR/PR/SD) at the landmark time were included in the analyses. Patients who received maintenance therapy after this time point were classified as the maintenance therapy group, whereas those who did not were classified as the active surveillance group.

PFS was defined as the time from the landmark date to disease progression or death from any cause. OS was calculated as the time from the landmark date to death or last follow-up. Patients without documented progression or death during follow-up were censored at the date of last contact.

### 2.4. Statistical Analysis

Statistical analyses were performed using IBM SPSS Statistics (version 28.0; IBM Corp., Armonk, NY, USA). During the development of the statistical analysis workflow, Claude (Anthropic, San Francisco, CA, USA), a large language model, was used to assist in coding strategy development, statistical syntax generation, and verification of analytical procedures. All statistical analyses, outputs, and reported results were independently executed and verified using IBM SPSS Statistics by the authors.

Continuous variables are expressed as mean ± standard deviation and median (minimum–maximum or Q1–Q3), while categorical variables are presented as frequencies and percentages (%). The distribution of variables was assessed using appropriate normality tests.

For comparisons between groups, continuous variables were analyzed using the independent samples *t*-test or the Mann–Whitney U test, as appropriate, while categorical variables were compared using the chi-square test or Fisher’s exact test.

Survival analyses were performed using the Kaplan–Meier method, and survival curves were compared using the log-rank test. Median potential follow-up duration was estimated using the reverse Kaplan–Meier method, and corresponding 95% confidence intervals were calculated.

Exploratory subgroup analyses were performed using Kaplan–Meier estimates and log-rank tests, with unadjusted hazard ratios and 95% confidence intervals estimated using Cox proportional hazards models. Treatment-by-subgroup interaction terms were used to assess potential heterogeneity of the association between maintenance therapy and survival outcomes. No adjustment for multiple comparisons was applied because these analyses were exploratory and hypothesis-generating.

Multivariable Cox proportional hazards regression models were constructed using a clinically defined adjustment set selected on the basis of established prognostic relevance and potential association with treatment selection rather than univariable *p*-value screening. The adjustment set included sex, comorbidity status, number of metastatic sites, primary tumor resection, treatment response, and distant lymph-node metastasis, together with maintenance therapy as the primary exposure. The proportional hazards assumption was assessed using Schoenfeld residuals.

Because this was a fixed retrospective cohort including all eligible patients, no formal sample-size or post hoc power calculation was performed; statistical precision was assessed using 95% confidence intervals around the effect estimates.

### 2.5. Ethical Approval

The study was conducted in accordance with the principles of the Declaration of Helsinki and was approved by the Ethics Committee of Istanbul University Istanbul Faculty of Medicine (approval date: 9 February 2026; approval number: 3904249). Due to the retrospective nature of the study, informed consent was waived.

## 3. Results

**Figure 1:**
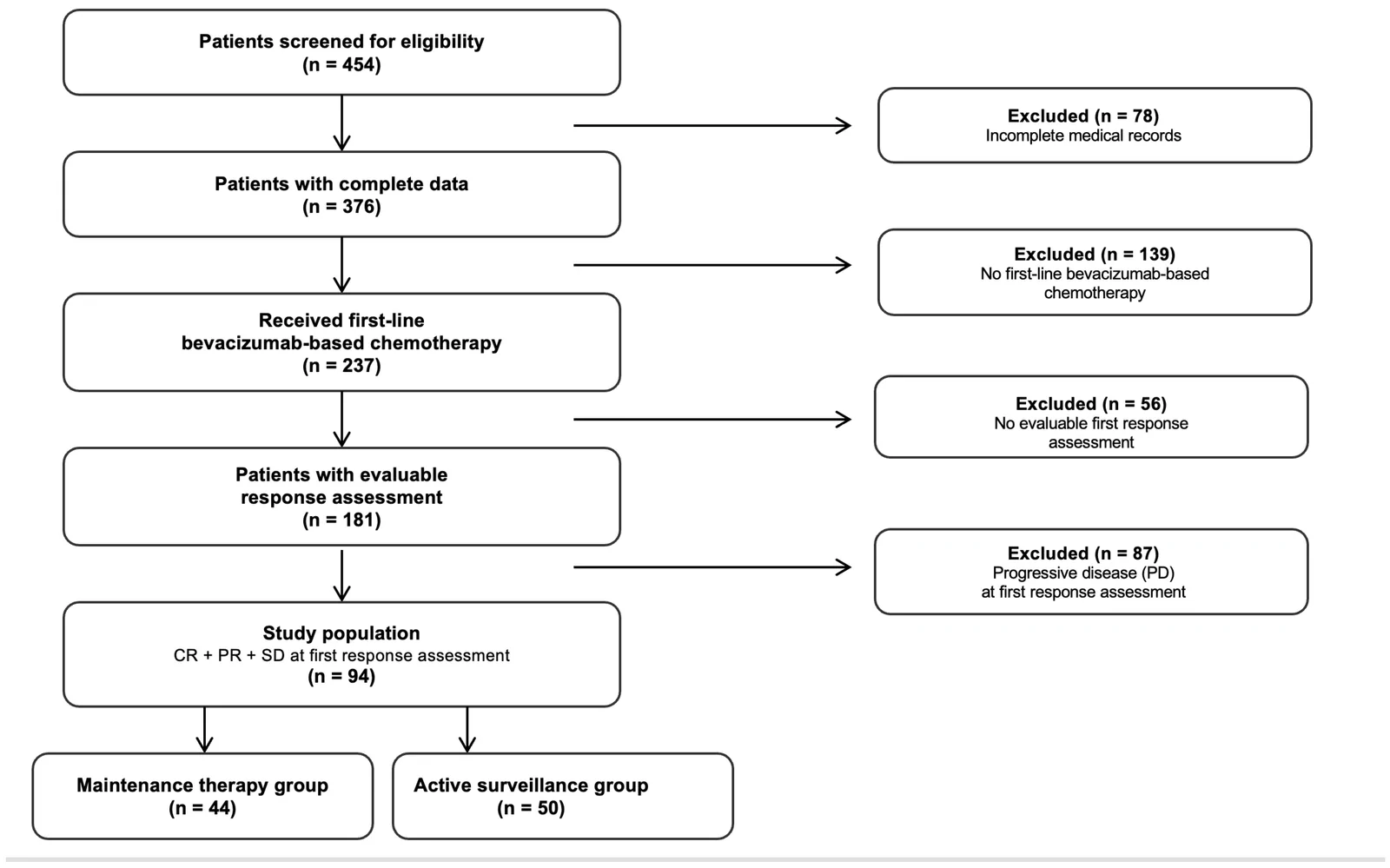
Study flow diagram.

A total of 454 patients were screened for eligibility. After exclusion of patients with incomplete medical records (n = 78), absence of first-line anti-VEGF-based therapy (n = 139), unavailable response assessment (n = 56), and progressive disease at the first response evaluation (n = 87), 94 patients who achieved disease control (CR/PR/SD) were included in the landmark cohort. Of these, 44 received maintenance therapy and 50 underwent active surveillance. Patients with unavailable response assessment did not return to our institution for the scheduled post-induction radiological evaluation, whereas patients with incomplete medical records lacked sufficient baseline clinicopathological, treatment, or follow-up information for reliable analysis. The patient selection process is illustrated in Figure 1.

### 3.1. Patient Characteristics

Of the 94 patients included in the overall cohort, the median age was 62 years (IQR: 55–71, range: 36–84, mean: 62.2 ± 10.6 years), and 58 patients (61.7%) were male. Overall, 44 patients (46.8%) received maintenance therapy, while 50 (53.2%) were managed in the active surveillance group. The median age was higher in the maintenance group compared to the surveillance group (65 vs. 60 years; *p* = 0.025). The proportion of patients aged ≥70 years was also significantly greater in the maintenance group (40.9% vs. 20.0%; *p* = 0.041).

No statistically significant differences were observed between the groups in terms of sex distribution, Eastern Cooperative Oncology Group performance status (ECOG PS), presence of comorbidities, tumor location, type of metastasis, number of metastatic sites, or RAS mutation status (Table 1).

A significant imbalance was observed regarding distant lymph-node metastasis, which was more frequent in the active surveillance group than in the maintenance group (26.0% vs. 6.8%, *p* = 0.015). Detailed clinical and pathological characteristics, including stage distribution, tumor marker levels, metastatic patterns, and comprehensive post-progression treatment data (second- and third-line treatment rates, chemotherapy regimens, biologic agents, treatment duration, and treatment response) are provided in Supplementary Table S1.

Patients in the maintenance group received a higher number of first-line chemotherapy cycles compared to those in the active surveillance group (10.8 vs. 9.1; *p* = 0.010). In addition, primary tumor resection was more frequently performed in the maintenance group (61.4% vs. 34.0%; *p* = 0.013). In contrast, metastasectomy during or after first-line treatment was more commonly performed in the active surveillance group (32.0% vs. 9.1%; *p* = 0.010) (Table 2).

Treatment-related adverse events are summarized in Supplementary Table S2. Any adverse event was more frequent in the maintenance group than in the active surveillance group (70.5% vs. 36.0%; *p* = 0.001), as were chemotherapy-related treatment modifications (38.6% vs. 16.0%; *p* = 0.019). The incidence of peripheral neuropathy was numerically higher in the maintenance group but did not reach statistical significance (31.8% vs. 16.0%; *p* = 0.089). The STROBE checklist for this study is provided in Supplementary Table S3.

### 3.2. Survival Analyses

The median potential follow-up duration was 75.2 months (95% CI: 41.6–92.8), as estimated using the reverse Kaplan–Meier method.

Median PFS was longer in the maintenance group compared to the active surveillance group (11.3 vs. 6.7 months); however, this difference did not reach statistical significance (log-rank *p* = 0.077). In contrast, median OS was significantly longer in the maintenance group (24.8 vs. 18.0 months) (log-rank *p* = 0.045).

**Figure 2:**
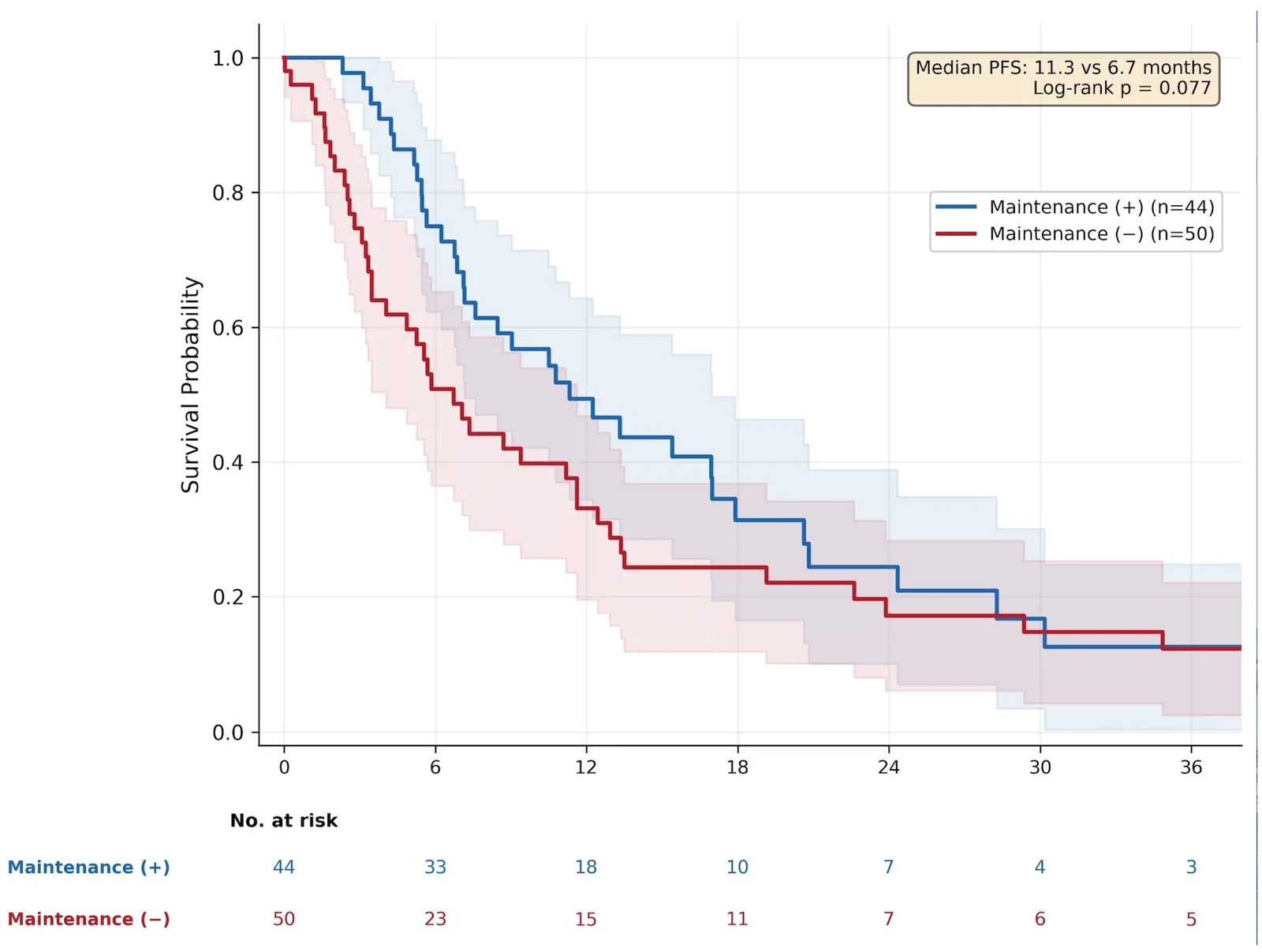
Kaplan–Meier Curves for Progression-Free Survival (PFS) According to Maintenance Therapy. Shaded areas represent 95% confidence intervals.

**Figure 3:**
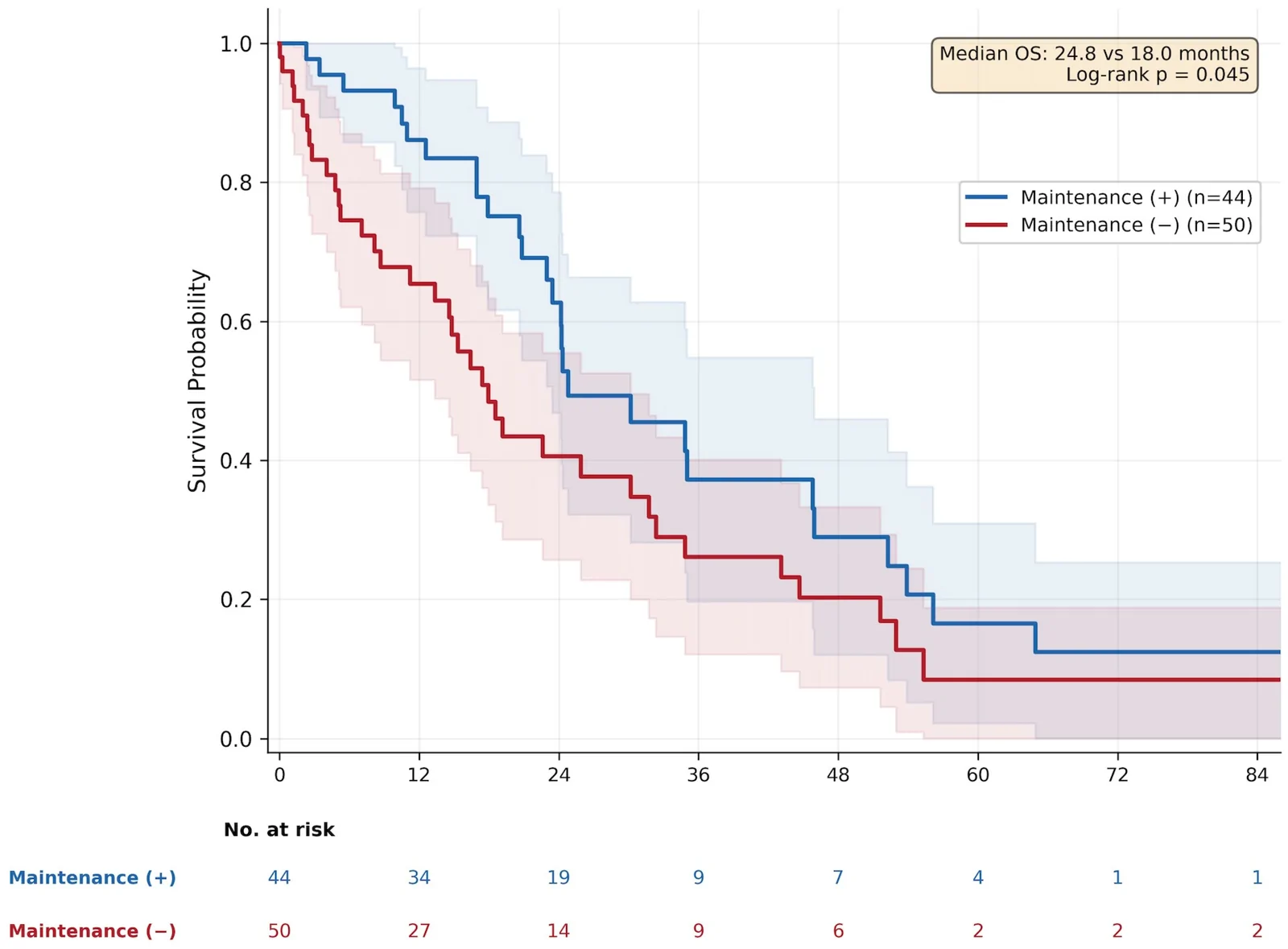
Kaplan–Meier Curves for Overall Survival (OS) According to Maintenance Therapy. Shaded areas represent 95% confidence intervals.

Kaplan–Meier survival curves are presented in Figure 2 (PFS) and Figure 3 (OS).

Survival rates also favored the maintenance group. The 12-month OS rate was 86.1% in the maintenance group compared to 65.4% in the active surveillance group, while the 24-month OS rates were 62.7% and 40.5%, respectively. Similarly, the 12-month PFS rate was 49.3% in the maintenance group and 33.1% in the active surveillance group (Table 3).

### 3.3. Subgroup Analyses

Exploratory subgroup analyses using time-to-event methods are presented in Table 4. For OS, maintenance therapy was associated with a lower hazard of death among patients with ≥2 metastatic sites (HR = 0.45, 95% CI: 0.22–0.88; log-rank *p* = 0.016). For PFS, lower hazards were observed among patients with RAS-mutant tumors (HR = 0.54, 95% CI: 0.30–0.99; *p* = 0.042), patients with comorbidities (HR = 0.50, 95% CI: 0.27–0.91; *p* = 0.019), and those with ≥2 metastatic sites (HR = 0.52, 95% CI: 0.28–0.99; *p* = 0.041). However, no treatment-by-subgroup interaction reached statistical significance, indicating no clear evidence of heterogeneity in the association between maintenance therapy and survival across the evaluated subgroup levels. Regimen-specific comparisons were also not statistically significant for either OS or PFS. Given the exploratory and hypothesis-generating nature of these analyses, no formal adjustment for multiple comparisons was applied, and the findings should therefore be interpreted cautiously.

### 3.4. Cox Regression Analyses

In the univariable analysis for OS, maintenance therapy was associated with a significant survival benefit (HR = 0.60; *p* = 0.047). Male sex (HR = 2.72; *p* < 0.001), the presence of ≥2 metastatic sites (HR = 2.57; *p* < 0.001), and distant lymph-node metastasis (HR = 2.10; *p* = 0.015) were associated with worse survival, whereas primary tumor resection (HR = 0.41; *p* = 0.001) and treatment response (CR + PR) (HR = 0.38; *p* < 0.001) were associated with improved survival.

In multivariable analysis, maintenance therapy was no longer independently associated with OS (HR = 0.68, 95% CI: 0.37–1.26; *p* = 0.220). Male sex (HR = 2.12; *p* = 0.019), presence of comorbidities (HR = 2.27; *p* = 0.009), and ≥2 metastatic sites (HR = 2.49; *p* = 0.006) remained independent adverse prognostic factors, whereas primary tumor resection (HR = 0.46; *p* = 0.022) and treatment response (HR = 0.36; *p* = 0.001) were independently associated with improved survival. Distant lymph-node metastasis showed a borderline association with worse OS (HR = 2.24; *p* = 0.056) (Table 5). No violation of the proportional hazards assumption was identified for the primary exposure variable (maintenance therapy) in either the univariable (Schoenfeld test, *p* = 0.494) or multivariable (*p* = 0.177) OS models.

In Cox regression analysis for PFS, maintenance therapy showed borderline significance in univariable analysis (HR = 0.66; *p* = 0.079) but did not remain independently associated with PFS in multivariable analysis (HR = 0.62, 95% CI: 0.36–1.07; *p* = 0.085). Presence of comorbidities remained independently associated with worse PFS (HR = 1.83; *p* = 0.027), whereas distant lymph-node metastasis was not significantly associated with PFS (HR = 1.30; *p* = 0.494) (Table 6). Similarly, the proportional hazards assumption was satisfied for maintenance therapy in the multivariable PFS model (Schoenfeld test, *p* = 0.356).

## 4. Discussion

This study provides real-world evidence on the association between maintenance therapy strategies and survival outcomes in patients with mCRC who achieved disease control following first-line bevacizumab combination chemotherapy. Consistent with findings from randomized clinical trials conducted in selected patient populations, maintenance therapy was associated with prolonged OS in this more heterogeneous real-world cohort. Although maintenance therapy was associated with longer unadjusted OS, this association was attenuated after adjustment for baseline prognostic factors, suggesting that part of the observed survival difference may have been influenced by imbalances in patient and disease characteristics between the treatment groups. By evaluating different maintenance approaches within a real-world cohort, our findings provide complementary evidence supporting the importance of treatment continuity in appropriately selected patients. It should also be noted that the Kaplan–Meier survival estimates presented in Table 3 represent unadjusted comparisons between groups. Given the baseline differences observed between treatment groups, including age distribution, primary tumor surgery, and treatment exposure, these findings should be interpreted in conjunction with the multivariable analyses, which account for measured prognostic factors and provide a more reliable estimate of the independent association between maintenance therapy and survival outcomes.

Randomized controlled trials and meta-analyses in the existing literature have consistently demonstrated that bevacizumab-based maintenance therapies significantly improve PFS in mCRC [3,4]. The magnitude of PFS benefit appears to increase with treatment intensity, with the combination of bevacizumab and a fluoropyrimidine (HR: 0.45) providing superior disease control compared to bevacizumab monotherapy (HR: 0.72) [4]. In this context, the CAIRO3 trial demonstrated that capecitabine plus bevacizumab significantly reduced the risk of progression compared to observation (HR: 0.40), while the AIO KRK 0207 study confirmed that maintenance therapy with a fluoropyrimidine plus bevacizumab resulted in superior PFS compared with bevacizumab monotherapy or observation [11,14].

Despite these consistent gains in PFS, the impact of maintenance therapy on OS has generally remained limited, often manifesting as a positive but statistically non-significant trend (HR: 0.89–0.93) in most studies [3,4]. However, key trials such as CAIRO3 and AIO KRK 0207 have highlighted that, in selected patient populations achieving an objective response to induction therapy (CR, PR), maintenance therapy may also confer a significant OS benefit [4,11,14]. In contrast, in the SAKK 41/06 trial, which compared bevacizumab monotherapy with observation, only a modest improvement in PFS (9.5 vs. 8.5 months; *p* = 0.025) was observed, without a statistically significant OS benefit, suggesting limited clinical value for single-agent bevacizumab in this setting [6].

When considering the generalizability of clinical trial data to routine practice, it is important to acknowledge that randomized trials are conducted in highly selected patient populations, typically comprising younger individuals with lower comorbidity burden and better performance status. In contrast, real-world mCRC populations are considerably more heterogeneous and often include patients with substantial comorbidity burden and variable treatment exposure. Compared with the landmark trials CAIRO3 and AIO KRK 0207, our study reflects routine clinical practice through a retrospective landmark design including a more heterogeneous population with a substantial burden of comorbidities [11,14]. These characteristics suggest that our findings may be more representative of patients encountered in daily oncology practice. In this context, as emphasized in the design of the PROMETCO study, the management of mCRC is increasingly conceptualized not as isolated lines of therapy but as a “continuum of care” model [13]. In our real-world cohort, maintenance therapy was associated with improved unadjusted survival outcomes. However, this association was no longer statistically significant after multivariable adjustment, highlighting the complexity of treatment selection in routine clinical practice. Consistent with the landmark trials, maintenance therapy was associated primarily with improved disease control rather than a robust independent survival benefit, although our observational design additionally demonstrated the influence of baseline prognostic differences on unadjusted survival outcomes [11,14]. The absence of a statistically significant PFS association after multivariable adjustment should be interpreted in the context of limited precision and residual clinical heterogeneity.

Furthermore, the management of cumulative toxicity—one of the key rationales underlying the transition to maintenance therapy—represents a critical determinant of clinical decision-making in routine practice. In particular, cumulative neurotoxicity associated with oxaliplatin-based regimens may significantly impair QoL and lead to premature discontinuation of treatment [1,7,15]. As demonstrated in the CONcePT trial, intermittent use of oxaliplatin or its omission during the maintenance phase can significantly improve time to treatment failure (TTF) [7]. Similarly, findings from the KSCC 0902 study support that bevacizumab-based maintenance strategies, with a favorable tolerability profile, prolong the duration of active treatment and may ultimately translate into cumulative survival benefits [5].

In the exploratory subgroup analyses, maintenance therapy was associated with longer PFS among patients with RAS-mutant tumors, whereas the corresponding OS association was not statistically significant. Importantly, treatment-by-RAS interaction tests were not statistically significant, providing no clear evidence that RAS status modified the association between maintenance therapy and survival. Therefore, these subgroup findings should be considered hypothesis-generating and interpreted cautiously. In the literature, RAS mutations are recognized as key biological markers associated with a more invasive disease course and a higher propensity for lung metastases in mCRC [16,17,18]. Mechanistically, RAS and VEGF signaling pathways are closely interconnected rather than completely independent. Constitutive activation of the RAS/MAPK pathway has been shown to upregulate VEGF expression and promote tumor angiogenesis [16,18]. Therefore, continued VEGF inhibition through maintenance therapy may remain clinically relevant in RAS-mutant tumors; however, the absence of a significant treatment-by-RAS interaction in our study precludes conclusions regarding a differential treatment effect according to RAS status.

Patients with comorbidities are typically underrepresented in randomized phase III trials, which predominantly enroll younger individuals with good performance status [13,19]. In our exploratory subgroup analysis, maintenance therapy was associated with longer PFS among patients with comorbidities, whereas the corresponding OS association did not reach statistical significance. Importantly, treatment-by-comorbidity interaction tests were not statistically significant, providing no clear evidence that comorbidity status modified the association between maintenance therapy and survival. Therefore, these findings should be interpreted cautiously and considered hypothesis-generating. Nevertheless, they support the value of real-world evidence in evaluating maintenance strategies in broader and more heterogeneous patient populations [13]. Current oncology guidelines recommend maintenance therapy with a fluoropyrimidine plus bevacizumab in patients who achieve disease control following induction therapy [1,20]. The AVEX trial demonstrated that the addition of bevacizumab significantly improved PFS (9.1 vs. 5.1 months) in elderly and frail patients who were not candidates for intensive chemotherapy [21]. Moreover, prospective cohort studies such as KSCC 0902 support the feasibility of bevacizumab-based strategies in older and more vulnerable patient populations encountered in routine clinical practice [5].

In patients with comorbid conditions, the primary rationale for transitioning to maintenance therapy lies in the management of cumulative toxicities associated with full-dose chemotherapy, particularly oxaliplatin-induced neurotoxicity [7]. The accumulation of such toxicities may compromise functional capacity and QoL, leading to early treatment discontinuation or dose reductions in this vulnerable population [21,22]. Maintenance strategies employing more tolerable agents, such as fluoropyrimidine ± bevacizumab, reduce the risk of grade 3/4 adverse events while maintaining biological pressure on the tumor, thereby delaying disease progression [3]. As demonstrated in the quality-of-life subgroup analyses of the AIO KRK 0207 trial, active maintenance approaches do not result in deterioration of global health status compared with treatment-free intervals; on the contrary, they contribute to stabilization of symptom control [14]. In this context, the PFS association observed among patients with comorbidities should be interpreted cautiously, particularly because the treatment-by-comorbidity interaction was not statistically significant.

In our study, the favorable impact of objective response to induction therapy (CR/PR) and primary tumor resection on survival outcomes is consistent with established prognostic determinants in mCRC. Landmark randomized trials such as CAIRO3 and AIO KRK 0207 have consistently demonstrated that patients achieving complete or partial response during induction derive significant overall survival benefits from subsequent maintenance strategies [4,11]. These findings support the notion that, in biologically treatment-sensitive tumors where substantial cytoreduction is achieved during initial therapy, continued maintenance pressure may maximize disease control and translate into improved cumulative survival outcomes [1,4]. Similarly, pulmonary metastasectomy has become an important component of multidisciplinary management in carefully selected patients with mCRC. When integrated with effective systemic therapy, it may contribute to durable disease control and prolonged survival [23].

Beyond therapeutic decision-making, recent advances in artificial intelligence, particularly deep learning-based computational pathology, have shown considerable promise in CRC diagnosis and tissue classification. Deep learning algorithms applied to digitized histopathological whole-slide images have demonstrated high accuracy in tissue classification, tumor detection, and the prediction of clinically relevant molecular features, including microsatellite instability, thereby supporting diagnostic standardization and reducing interobserver variability. Although these technologies were beyond the scope of the present study, their integration with clinical, pathological, and treatment-related variables may further improve patient stratification and enable more individualized therapeutic approaches in future CRC research [24,25,26]. Similarly, emerging serum biomarkers such as butyrylcholinesterase have recently attracted interest as potential prognostic indicators in colorectal cancer. Lower cholinesterase levels have been associated with poorer survival outcomes, suggesting that the integration of novel biochemical biomarkers with clinicopathological variables and artificial intelligence-based predictive models may further improve individualized risk stratification in future studies [27].

In addition to advances in systemic treatment, colorectal cancer screening remains one of the most effective strategies for reducing disease incidence and mortality through the detection and removal of premalignant lesions and the diagnosis of early-stage disease. Adherence to evidence-based screening recommendations should be promoted not only in the general population but also among healthcare professionals, who play a key role in patient education and the implementation of preventive strategies. Although the present study focused on the management of metastatic disease, improving participation in screening programs remains essential for reducing the future burden of colorectal cancer [28,29].

When interpreting the findings of this study, both its strengths and limitations should be considered. One of the major strengths of the study is its reliance on real-world data, which enables the inclusion of a more heterogeneous patient population characterized by older age and a higher burden of comorbidities, thereby enhancing the generalizability of the findings to routine clinical practice. In addition, the use of a landmark analysis to minimize immortal time bias represents an important methodological strength. Furthermore, the use of multivariable analyses allowed adjustment for important prognostic factors, providing a more comprehensive assessment of the association between maintenance therapy and survival outcomes.

However, several limitations should also be acknowledged. First, the retrospective and single-center design inherently introduces the potential for selection bias and the influence of unmeasured confounding factors. In real-world practice, the decision to initiate maintenance therapy was not randomized but was based on physician judgment and patient preference while generally reflecting the contemporaneous institutional practice, emerging evidence, and international guideline recommendations throughout the study period. The observed baseline differences likely reflect treatment selection in routine clinical practice rather than a direct effect of maintenance therapy itself. Therefore, residual confounding related to treatment selection, including differences in surgical management such as primary tumor resection and metastasectomy, cannot be completely excluded despite multivariable adjustment. Patients who underwent primary tumor resection, received a greater number of induction chemotherapy cycles, or had a lower burden of adverse disease characteristics may have been considered more suitable candidates for continued treatment. Multivariable Cox regression models were constructed using a clinically defined adjustment set based on established prognostic relevance and potential association with treatment selection. However, because of the relatively small sample size and limited number of outcome events, not all baseline characteristics that differed between groups (including age, surgical intent, metastasectomy, and number of first-line chemotherapy cycles) could be incorporated into the final multivariable models. Furthermore, molecular variables such as BRAF mutation and MSI status were not included in the multivariable analyses because these data were unavailable for a substantial proportion of patients, and their inclusion would have considerably reduced the effective sample size. In addition, comorbidity was assessed as a binary variable rather than with a validated comorbidity index, limiting characterization of the number, type, and severity of coexisting conditions. Notably, the association between maintenance therapy and overall survival was attenuated after adjustment, suggesting that baseline differences may have contributed to the survival advantage observed in the unadjusted analyses. Furthermore, although propensity score-based methods, such as propensity score matching or inverse probability weighting, may reduce treatment-selection bias in observational studies, they were not applied because of the relatively small sample size and limited number of outcome events, which could have resulted in substantial loss of effective sample size after matching or unstable estimates with inverse probability weighting. Therefore, treatment-selection bias was addressed using multivariable Cox regression models adjusted for a clinically defined set of prognostic factors. Consequently, the results should be interpreted with caution, as residual confounding related to baseline imbalances cannot be completely excluded.

Second, the relatively limited sample size resulted in reduced precision, particularly in subgroup analyses, which may explain why some clinically meaningful differences did not reach statistical significance. In addition, no formal correction for multiple comparisons was applied to the exploratory subgroup analyses. Therefore, these findings should be considered hypothesis-generating and interpreted cautiously because of the increased risk of false-positive results. Additionally, the heterogeneity of maintenance regimens (e.g., capecitabine, bevacizumab-based combinations) limits the ability to draw definitive conclusions regarding the superiority of specific treatment strategies. Regimen-specific adjusted analyses could not be reliably performed because of the small number of patients and outcome events within each maintenance subgroup; therefore, the regimen-specific comparisons remain exploratory and susceptible to confounding. Because this study was conducted over a 10-year period, temporal changes in institutional practice should be considered when interpreting the findings. At our institution, the indications and contraindications for fluoropyrimidine-based combination chemotherapy remained largely unchanged throughout the study period. Similarly, surveillance protocols remained consistent, consisting of approximately three-monthly clinical evaluation, laboratory assessment, and contrast-enhanced computed tomography. The principal change over time was the increasing adoption of fluoropyrimidine ± bevacizumab maintenance therapy in patients achieving disease control after induction treatment, reflecting the incorporation of emerging evidence and guideline recommendations into routine clinical practice. Accordingly, temporal changes in institutional practice may have influenced both treatment allocation and survival outcomes and therefore cannot be completely excluded as a source of residual confounding. QoL represents an important clinical objective of maintenance therapy, aiming to preserve treatment benefit while minimizing cumulative toxicity. Finally, due to the retrospective nature of data collection, QoL outcomes could not be assessed using standardized instruments, precluding a comprehensive evaluation of the functional and patient-reported impact of maintenance therapy. Despite these limitations, our study provides clinically relevant evidence by reflecting real-world practice and incorporating robust methodological approaches such as landmark analysis, thereby contributing valuable data to inform treatment strategies in mCRC.

These real-world data suggest a potential survival advantage associated with maintenance therapy following first-line bevacizumab combination chemotherapy in patients with mCRC who achieve disease control. However, this association was observed only in the unadjusted analyses and was not maintained after multivariable adjustment, suggesting that baseline differences may have contributed to the observed findings.

## Figures and Tables

**Table 1:** Baseline Characteristics of Patients.

Variable	Anti-VEGF Maintenance (+) (n = 44)	Anti-VEGF Maintenance (−) (n = 50)	*p*-Value
**DEMOGRAPHIC CHARACTERISTICS**			
**Age (years)**			
Mean ± SD	64.8 ± 10.6	59.9 ± 10.2	**0.025 ^m^**
Median (Min–Max)	65 (36–84)	60 (36–79)	
**Age Group**			
<65 years	21 (47.7%)	34 (68.0%)	0.060 ^X2^
≥65 years	23 (52.3%)	16 (32.0%)	
<70 years	26 (59.1%)	40 (80.0%)	**0.041 ^X2^**
≥70 years	18 (40.9%)	10 (20.0%)	
**Sex**			
Female	18 (40.9%)	18 (36.0%)	0.674 ^X2^
Male	26 (59.1%)	32 (64.0%)	
**BMI (kg/m^2^)**			
Mean ± SD	25.0 ± 4.9	26.7 ± 4.7	0.107 ^m^
Median (Min–Max)	25.0 (13.3–36.6)	26.2 (19.3–44.0)	
**ECOG Performance Status**			
ECOG 0	35 (79.5%)	39 (78.0%)	0.519 ^X2^
ECOG 1	8 (18.2%)	11 (22.0%)	
ECOG 2	1 (2.3%)	0 (0.0%)	
**Presence of Comorbidity**	24 (58.5%)	25 (52.1%)	0.669 ^X2^
**TUMOR AND PATHOLOGICAL CHARACTERISTICS**			
**Tumor Location**			
Right colon	13 (29.5%)	12 (24.0%)	0.642 ^X2^
Left colon	31 (70.5%)	38 (76.0%)	
**Histological Type**			
Adenocarcinoma NOS	40 (90.9%)	43 (86.0%)	0.434 ^X2^
Special Histological Subtype	4 (9.1%)	7 (14.0%)	
**Primary Tumor Surgery**			
Performed	27 (61.4%)	17 (34.0%)	**0.013 ^X2^**
Not performed	17 (38.6%)	33 (66.0%)	
**Surgical Intent**			
Curative	8 (18.2%)	7 (14.0%)	**0.022 ^X2^**
Palliative	19 (43.2%)	10 (20.0%)	
No surgery performed	17 (38.6%)	33 (66.0%)	
**RAS Status**			
Wild-type	12 (29.3%)	15 (35.7%)	
Mutant	29 (70.7%)	27 (64.3%)	0.641 ^X2^
**METASTATIC CHARACTERISTICS**			
**Metastasis Type**			
Synchronous	36 (81.8%)	42 (84.0%)	0.791 ^X2^
Metachronous	8 (18.2%)	8 (16.0%)	
**Number of Metastatic Sites**			
Mean ± SD	1.5 ± 0.7	1.7 ± 0.9	0.396 ^m^
1 site(s)	25 (56.8%)	26 (52.0%)	
2 site(s)	16 (36.4%)	15 (30.0%)	
3 site(s)	2 (4.5%)	7 (14.0%)	
4 site(s)	1 (2.3%)	2 (4.0%)	

^m^ Independent samples Mann–Whitney U test. ^X2^ Chi-square test/Fisher exact test. *p* < 0.05 was considered statistically significant.

**Table 2:** First-Line Treatment and Maintenance Characteristics.

Variable	Anti-VEGF Maintenance (+) (n = 44)	Anti-VEGF Maintenance (−) (n = 50)	*p*-Value
**METASTATIC FIRST-LINE TREATMENT (Total n = 94)**			
**First-Line Chemotherapy Regimen**	**n = 44**	**n = 50**	0.213 ^X2^
CAPOX	17 (38.6%)	14 (28.0%)	
CAPIRI	0 (0.0%)	2 (4.0%)	
FOLFOX	22 (50.0%)	23 (46.0%)	
FOLFIRI	3 (6.8%)	4 (8.0%)	
Capecitabine + Oxaliplatin + Irinotekan	2 (4.5%)	2 (4.0%)	
FOLFOXIRI	0 (0.0%)	5 (10.0%)	
**First-Line Biologic Agent**	Bevacizumab: 100%	Bevacizumab: 100%	
**First-Line Number of Cycles**			
Mean ± SD	10.8 ± 3.0	9.1 ± 3.9	**0.010 ^m^**
**First-Line Treatment Response**	**n = 44**	**n = 50**	0.511 ^X2^
Complete Response (CR)	8 (18.2%)	7 (14.0%)	
Partial Response (PR)	28 (63.6%)	29 (58.0%)	
Stable Disease (SD)	8 (18.2%)	14 (28.0%)	
ORR (CR + PR)	36 (81.8%)	36 (72.0%)	
**Metastasectomy During/After 1L Treatment**			
Yes	4 (9.1%)	16 (32.0%)	**0.010 ^X2^**
No	40 (90.9%)	34 (68.0%)	
**Surgical Outcome**	n = 4	n = 16	
Complete resection (R0)	1 (25.0%)	7 (43.8%)	
Residual disease (R+)	3 (75.0%)	9 (56.2%)	
**FIRST-LINE MAINTENANCE TREATMENT**			
**Maintenance Treatment Content**	**n = 44**	**-**	
Capecitabine	15 (34.1%)	-	
Capecitabine + Bevacizumab	12 (27.3%)	-	
5-fluorouracil/leucovorin (5-FU/LV) plus bevacizumab	17 (38.6%)	-	
**Maintenance Treatment Duration (Months)**			
Mean ± SD	6.5 ± 4.4	-	
Second Maintenance Treatment (Switch)	2 (4.5%)	-	
Capecitabine	2 (100%)	-	
**Second Maintenance Treatment Duration (Months)**			
Patient 1: Capecitabine + Bev → Capecitabine	30 months	-	
Patient 2: 5-FU/LV + Bev → Capecitabine	36 months	-	
**TREATMENT LINE TRANSITION SUMMARY**			
Received ≥2 Lines of Treatment	23 (52.3%)	22 (44.0%)	
Received ≥3 Lines of Treatment	16 (36.4%)	10 (20.0%)	

^m^ Independent samples Mann–Whitney U test. ^X2^ Chi-square test/Fisher exact test. *p* < 0.05 was considered statistically significant.

**Table 3:** Progression-Free Survival (PFS) and Overall Survival (OS) Outcomes According to Maintenance Therapy.

Variable	Maintenance (+) (n = 44)	Maintenance (−) (n = 50)	*p*-Value
**PROGRESSION-FREE SURVIVAL (PFS)**			
No. of Patients Evaluated	44	50	
No. of Events (Progression/Death)	33	43	
No. of Censored Patients	11	7	
Median PFS (Months)	11.3	6.7	0.077 ^K^
95% Confidence Interval	7.2–17.0	4.0–11.6	
6-Month PFS Rate	75.0%	50.8%	
12-Month PFS Rate	49.3%	33.1%	
24-Month PFS Rate	24.4%	17.2%	
Unadjusted HR (95% CI)	0.66 (0.42–1.05)		0.079 ^c^
Adjusted HR (95% CI) †	0.62 (0.36–1.07)		0.085 ^c^
**OVERALL SURVIVAL (OS)**			
No. of Patients Evaluated	44	50	
No. of Events (Death)	27	36	
No. of Censored Patients	17	14	
Median OS (Months)	24.8	18.0	**0.045 ^K^**
95% Confidence Interval	23.5–46.0	13.4–30.2	
12-Month OS Rate	86.1%	65.4%	
24-Month OS Rate	62.7%	40.5%	
36-Month OS Rate	37.2%	26.1%	
60-Month OS Rate	16.5%	8.4%	
Unadjusted HR (95% CI)	0.60 (0.36–0.99)		0.047 ^c^
Adjusted HR (95% CI) †	0.68 (0.37–1.26)		0.220 ^c^

^K^ Kaplan–Meier survival analysis with log-rank test, calculated from the landmark date. † Adjusted for sex, comorbidity, synchronous metastasis, number of metastatic sites, primary surgery, treatment response, and distant lymph-node metastasis, ^c^ Cox proportional hazards regression. HRs represent the effect of maintenance therapy versus active surveillance. Unadjusted HRs were estimated using univariable Cox proportional hazards regression; adjusted HRs were obtained from the multivariable Cox models using the clinically defined adjustment set described in the Statistical Analysis Section.

**Table 4:** Comparison of Observed OS and PFS Durations According to Maintenance Therapy Within Clinical Subgroups.

**OVERALL SURVIVAL (OS)**							
**Subgroup**	**n(+)**	**n(−)**	**mOS** **Maint. (+)** **(Months)**	**mOS** **Maint. (−)** **(Months)**	**HR (95% CI)**	***p*-Value**	**p-Interaction**
RAS Wild-Type	12	15	23.5	30.2	0.69 (0.29–1.64)	0.364 ^K^	0.703 ^c^
RAS Mutant	29	27	30.2	16.4	0.61 (0.31–1.19)	0.136 ^K^	
Right Colon	13	12	30.2	22.6	0.56 (0.23–1.38)	0.192 ^K^	0.965 ^c^
Left Colon	31	38	24.8	17.4	0.59 (0.32–1.08)	0.080 ^K^	
Age < 65	21	34	35.0	16.4	0.55 (0.27–1.12)	0.094 ^K^	0.917 ^c^
Age ≥ 65	23	16	24.8	19.2	0.59 (0.28–1.23)	0.141 ^K^	
Age < 70	26	40	24.8	16.4	0.57 (0.30–1.08)	0.078 ^K^	0.783 ^c^
Age ≥ 70	18	10	24.3	32.4	0.65 (0.27–1.58)	0.321 ^K^	
With Comorbidity	24	25	24.2	17.4	0.54 (0.28–1.03)	0.051 ^K^	0.502 ^c^
Without Comorbidity	17	23	45.8	18.0	0.72 (0.31–1.64)	0.430 ^K^	
Synchronous	36	42	24.2	18.0	0.63 (0.37–1.08)	0.090 ^K^	0.689 ^c^
Metachronous	8	8	24.8	32.4	0.57 (0.15–2.13)	0.396 ^K^	
Single Met. Site	25	26	35.0	34.9	0.85 (0.41–1.79)	0.668 ^K^	0.150 ^c^
Multiple Met. Sites (≥2)	19	24	23.5	7.1	0.45 (0.22–0.88)	0.016 ^K^	
Distant LN (+)	3	13	17.0	4.8	0.50 (0.11–2.25)	0.349 ^K^	0.742 ^c^
Distant LN (−)	41	37	24.8	22.6	0.69 (0.39–1.21)	0.187 ^K^	
**BY MAINTENANCE CONTENT**							
Capecitabine vs. None	15	50	34.9	18.0	0.68 (0.34–1.33)	0.248 ^K^	
Capecitabine+Bev vs. None	12	50	30.2	18.0	0.51 (0.20–1.30)	0.149 ^K^	
5-FU/LV+Bev vs. None	17	50	24.2	18.0	0.62 (0.32–1.22)	0.164 ^K^	
**PROGRESSION-FREE SURVIVAL (PFS)**							
**Subgroup**	**n(+)**	**n(−)**	**mPFS** **Maint.(+)** **(months)**	**mPFS** **Maint.(−)** **(months)**	**HR (95% CI)**	***p*-value**	**p-interaction**
RAS Wild-Type	12	15	7.1	13.5	1.36 (0.62–2.95)	0.428 ^K^	0.076 ^c^
RAS Mutant	29	27	11.3	5.7	0.54 (0.30–0.99)	0.042 ^K^	
Right Colon	13	12	10.5	7.1	0.73 (0.31–1.68)	0.439 ^K^	0.700 ^c^
Left Colon	31	38	15.4	5.8	0.62 (0.36–1.07)	0.079 ^K^	
Age < 65	21	34	15.4	5.8	0.68 (0.36–1.27)	0.213 ^K^	0.759 ^c^
Age ≥ 65	23	16	10.8	9.4	0.63 (0.32–1.25)	0.182 ^K^	
Age < 70	26	40	12.3	5.7	0.60 (0.34–1.07)	0.077 ^K^	0.723 ^c^
Age ≥ 70	18	10	10.8	11.2	0.79 (0.35–1.82)	0.582 ^K^	
With Comorbidity	24	25	11.3	5.6	0.50 (0.27–0.91)	0.019 ^K^	0.176 ^c^
Without Comorbidity	17	23	12.3	9.4	0.86 (0.42–1.78)	0.683 ^K^	
Synchronous	36	42	10.5	5.8	0.74 (0.46–1.21)	0.224 ^K^	0.602 ^c^
Metachronous	8	8	24.3	12.9	0.54 (0.15–2.03)	0.353 ^K^	
Single Met. Site	25	26	11.3	11.6	0.84 (0.44–1.60)	0.595 ^K^	0.309 ^c^
Multiple Met. Sites (≥2)	19	24	12.3	3.4	0.52 (0.28–0.99)	0.041 ^K^	
Distant LN (+)	3	13	17.0	2.8	0.53 (0.12–2.35)	0.371 ^K^	0.613 ^c^
Distant LN (−)	41	37	11.3	7.4	0.75 (0.46–1.24)	0.264 ^K^	
**BY MAINTENANCE CONTENT**							
Capecitabine vs. None	15	50	12.3	6.7	0.74 (0.38–1.44)	0.371 ^K^	
Capecitabine + Bev vs. None	12	50	8.5	6.7	0.65 (0.32–1.34)	0.238 ^K^	
5-FU/LV + Bev vs. None	17	50	10.5	6.7	0.65 (0.35–1.20)	0.162 ^K^	

^K^ Kaplan–Meier analysis with log-rank test; ^c^ Cox regression for maintenance; mOS and mPFS are Kaplan–Meier median estimates from the landmark date. HRs are unadjusted for maintenance versus active surveillance (HR < 1 favors maintenance). All subgroup analyses were exploratory, with no adjustment for multiple comparisons.

**Table 5:** Univariable and Multivariable Cox Regression Analyses for Overall Survival (OS).

Overall Survival (OS)			
Univariate Analysis	HR	95% CI	*p*-Value
Maintenance Treatment (Yes vs. No)	0.60	0.36–0.99	**0.047 ^c^**
Age (≥65 vs. <65)	1.17	0.71–1.93	0.534 ^c^
Age (≥70 vs. <70)	0.92	0.55–1.56	0.768 ^c^
Sex (Male vs. Female)	2.72	1.53–4.83	**<0.001 ^c^**
ECOG PS (≥1 vs. 0)	1.10	0.61–1.97	0.754 ^c^
Comorbidity (Present vs. Absent)	1.66	0.98–2.80	0.058 ^c^
Tumor Location (Left vs. Right)	0.89	0.52–1.53	0.684 ^c^
RAS (Mutant vs. Wild-Type)	1.02	0.60–1.75	0.938 ^c^
Metastasis (Synchronous vs. Metachronous)	1.67	0.82–3.41	0.158 ^c^
Metastatic Sites (≥2 vs. 1)	2.57	1.56–4.25	**<0.001 ^c^**
Primary Surgery (Yes vs. No)	0.41	0.24–0.70	**0.001 ^c^**
Response (CR + PR vs. SD)	0.38	0.21–0.66	**<0.001 ^c^**
Distant LN Metastasis (Yes vs. No)	2.10	1.16–3.83	**0.015 ^c^**
**Multivariate Analysis (n = 89)**	**HR**	**95% CI**	***p*-value**
Maintenance Treatment (Yes vs. No)	0.68	0.37–1.26	0.220 ^c^
Sex (Male vs. Female)	2.12	1.13–3.98	**0.019 ^c^**
Comorbidity (Present vs. Absent)	2.27	1.22–4.23	**0.009 ^c^**
Metastasis (Synchronous vs. Metachronous)	0.90	0.38–2.11	0.812 ^c^
Metastatic Sites (≥2 vs. 1)	2.49	1.30–4.77	**0.006 ^c^**
Primary Surgery (Yes vs. No)	0.46	0.23–0.89	**0.022 ^c^**
Response (CR + PR vs. SD)	0.36	0.19–0.66	**0.001 ^c^**
Distant LN Metastasis (Yes vs. No)	2.24	0.98–5.12	0.056 ^c^

^c^ Cox proportional hazards regression (from landmark date). Univariate *p* < 0.25 → Included in multivariate. *p* < 0.05 significant.

**Table 6:** Univariable and Multivariable Cox Regression Analyses for Progression-Free Survival (PFS).

Progression-Free Survival (PFS)			
Univariate Analysis	HR	95% CI	*p*-Value
Maintenance Treatment (Yes vs. No)	0.66	0.42–1.05	0.079 ^c^
Age (≥65 vs. <65)	1.07	0.67–1.69	0.783 ^c^
Age (≥70 vs. <70)	0.94	0.58–1.54	0.819 ^c^
Sex (Male vs. Female)	1.73	1.04–2.86	**0.034 ^c^**
ECOG PS (≥1 vs. 0)	1.40	0.82–2.37	0.217 ^c^
Comorbidity (Present vs. Absent)	1.51	0.94–2.43	0.085 ^c^
Tumor Location (Left vs. Right)	0.85	0.52–1.40	0.523 ^c^
RAS (Mutant vs. Wild-Type)	1.10	0.67–1.80	0.706 ^c^
Metastasis (Synchronous vs. Metachronous)	2.25	1.11–4.58	**0.025 ^c^**
Metastatic Sites (≥2 vs. 1)	1.78	1.13–2.80	**0.013 ^c^**
Primary Surgery (Yes vs. No)	0.54	0.34–0.86	**0.009 ^c^**
Response (CR + PR vs. SD)	0.50	0.30–0.85	**0.010 ^c^**
Distant LN Metastasis (Yes vs. No)	1.56	0.88–2.76	0.125 ^c^
**Multivariate Analysis (n = 89)**	**HR**	**95% CI**	***p*-value**
Maintenance Treatment (Yes vs. No)	0.62	0.36–1.07	0.085 ^c^
Sex (Male vs. Female)	1.31	0.77–2.23	0.321 ^c^
ECOG PS (≥1 vs. 0)	1.31	0.73–2.35	0.356 ^c^
Comorbidity (Present vs. Absent)	1.83	1.07–3.13	**0.027 ^c^**
Metastasis (Synchronous vs. Metachronous)	1.39	0.63–3.08	0.419 ^c^
Metastatic Sites (≥2 vs. 1)	1.65	0.90–3.01	0.104 ^c^
Primary Surgery (Yes vs. No)	0.74	0.42–1.30	0.293 ^c^
Response (CR + PR vs. SD)	0.58	0.33–1.04	0.068 ^c^
Distant LN Metastasis (Yes vs. No)	1.30	0.61–2.74	0.494 ^c^

^c^ Cox proportional hazards regression (from landmark date). Univariate *p* < 0.25 → Included in multivariate. *p* < 0.05 significant.

## Data Availability

The datasets used and/or analyzed during the current study are available from the corresponding author on reasonable request.

## Supplementary Materials

The following supporting information can be downloaded at: https://www.mdpi.com/article/10.3390/jcm15166279/s1, Table S1: Detailed Clinical, Pathological, and Treatment Characteristics; Table S2: Treatment-Related Adverse Events During First-Line Therapy According to Subsequent Treatment Strategy; Figure S1: Forest plot for overall survival across subgroups (landmark analysis); Figure S2: Forest plot for progression-free survival across subgroups (landmark analysis); Table S3: STROBE Checklist.
