# Supplementary material for: Association of Maintenance Therapy Strategies with Survival Following First-Line Bevacizumab-Based Combination Chemotherapy in Metastatic Colorectal Cancer: A Single-Center Retrospective Observational Study
Source: J Clin Med. 2026 Aug 13;15(16):6279. doi: 10.3390/jcm15166279 (PMC13513069; doi:10.3390/jcm15166279)
Supplement: Supplementary file 1 [file jcm-15-06279-s001.zip › STROBE_Checklist_Filled Table S3.pdf]

STROBE Statement — Checklist of items that should be included in reports of observational studies (Cohort Study)

Manuscript: "Association of Maintenance Therapy Strategies with Survival Following First-Line Bevacizumab-Based Combination Chemotherapy in Metastatic Colorectal Cancer: A Single-Center Retrospective Observational Study" — Çokgezer, Şakar, Karabulut

Section	Item No.	Recommendation	Page No.	Relevant text from manuscript
Title and abstract	**1 (a)**	Indicate the study's design with a commonly used term in the title or the abstract	1, 3	Title: "...A Single-Center Retrospective Observational Study." Abstract (Methods): "This retrospective study included 94 mCRC patients..."
	**(b)**	Provide in the abstract an informative and balanced summary of what was done and what was found	3	Structured abstract (Objectives/Methods/Results/Conclusion) reporting the landmark cohort (n=94; maintenance n=44 vs. surveillance n=50), the OS benefit that did not persist after adjustment (median OS 24.8 vs 18.0 months, p=0.045; multivariable HR not significant), and PFS results (11.3 vs 6.7 months, p=0.077).
**Introduction**				
Background/rationale	**2**	Explain the scientific background and rationale for the investigation being reported	3–5	Introduction paragraphs on CRC epidemiology, first-line bevacizumab-based therapy, the induction-and-maintenance strategy, existing RCT evidence (CAIRO3, AIO KRK 0207), and the limited generalisability of RCTs to real-world practice, concluding with the rationale for real-world evidence (RWE).
Objectives	**3**	State specific objectives, including any prespecified hypotheses	5	"In this retrospective real-world observational study, we aimed to evaluate the association between maintenance therapy and survival outcomes...We hypothesized that maintenance therapy would be associated with survival outcomes after adjustment for clinically relevant prognostic factors in a heterogeneous real-world population."
**Methods**				
Study design	**4**	Present key elements of study design early in the paper	5	"Study Design and Patient Selection": "This study was designed as a single-center, retrospective, observational analysis to evaluate the association between maintenance therapy and survival outcomes..."
Setting	**5**	Describe the setting, locations, and relevant dates, including periods of recruitment, exposure, follow-up, and data collection	5	Single institution (Department of Medical Oncology, Institute of Oncology, Istanbul University); patients diagnosed January 2015–December 2025; data retrospectively extracted from electronic medical records between March and May 2026; data cutoff December 31, 2025.
Participants	**6 (a)**	Cohort study—Give the eligibility criteria, and the sources and methods of selection of participants. Describe methods of follow-up	5–6	Inclusion: age ≥18 years, histopathologically confirmed mCRC, first-line bevacizumab-based combination chemotherapy with disease control (CR/PR/SD); exclusion: primary progressive disease, incomplete records, unavailable response assessment. Follow-up via regular clinical/radiological assessment (≈every 3 months); landmark date defined at first post-induction response evaluation (≈p.7–8).

Section	Item No.	Recommendation	Page No.	Relevant text from manuscript
	**(b)**	Cohort study—For matched studies, give matching criteria and number of exposed and unexposed	—	Not applicable — this is not a matched cohort study. Groups were defined by treatment strategy (maintenance, n=44; active surveillance, n=50), not by matching.
Variables	**7**	Clearly define all outcomes, exposures, predictors, potential confounders, and effect modifiers. Give diagnostic criteria, if applicable	6	"Assessment of Clinical and Treatment Characteristics": age, sex, performance status, tumor location, metastatic burden/distribution, surgical status, chemotherapy regimens, anti-VEGF/maintenance status, comorbidity, RAS/BRAF/MSI status. Exposure = maintenance therapy vs. active surveillance; outcomes = PFS and OS (defined on p.8).
Data sources/ measurement	**8***	For each variable of interest, give sources of data and details of methods of assessment (measurement). Describe comparability of assessment methods if there is more than one group	6	Data obtained from institutional electronic medical records (and outside pathology/molecular reports when available); response assessed by contrast-enhanced CT per routine institutional protocol — identical assessment schedule (≈every 3 months) applied to both groups (p.7).
Bias	**9**	Describe any efforts to address potential sources of bias	7, 9, 17	Landmark analysis approach used "to minimize immortal time bias" (p.7); multivariable Cox regression adjusted for a clinically defined confounder set (p.9); limitations acknowledge residual confounding from non-randomized treatment selection despite adjustment (p.17).
Study size	**10**	Explain how the study size was arrived at	9	"Because this was a fixed retrospective cohort including all eligible patients, no formal sample-size or post hoc power calculation was performed; statistical precision was assessed using 95% confidence intervals." All eligible patients meeting criteria during the study period were included (p.6).
Quantitative variables	**11**	Explain how quantitative variables were handled in the analyses. If applicable, describe which groupings were chosen and why	8, 10	Continuous variables reported as mean±SD or median (range/IQR) with normality testing (p.8); age was additionally dichotomized at ≥65 and ≥70 years for descriptive/subgroup comparison (Table 1, p.10/24).
Statistical methods	**12 (a)**	Describe all statistical methods, including those used to control for confounding	8–9	t-test/Mann–Whitney U and chi-square/Fisher's exact test for group comparisons; Kaplan–Meier method with log-rank test; multivariable Cox proportional-hazards regression adjusted for sex, comorbidity, number of metastatic sites, primary tumor resection, treatment response, and distant lymph-node metastasis, to control for confounding of the maintenance-therapy exposure.
	**(b)**	Describe any methods used to examine subgroups and interactions	8	"Exploratory subgroup analyses were performed using Kaplan–Meier estimates and log-rank tests, with unadjusted HRs...Treatment-by-subgroup interaction terms were used to assess potential heterogeneity..."
	**(c)**	Explain how missing data were addressed	5	"Complete baseline clinicopathological characteristics, treatment information, radiological response assessment, and follow-up data were required for study inclusion"; patients with incomplete records (n=78) were excluded (p.9) — i.e., a complete-case cohort; no imputation was performed/reported.

Section	Item No.	Recommendation	Page No.	Relevant text from manuscript
	**(d)**	Cohort study—If applicable, explain how loss to follow-up was addressed	8	"Patients without documented progression or death during follow-up were censored at the date of last contact." Median potential follow-up (75.2 months) estimated by the reverse Kaplan–Meier method (p.10).
	**(e)**	Describe any sensitivity analyses	—	Not explicitly reported as a separate sensitivity analysis. The manuscript notes proportional-hazards assumption checks via Schoenfeld residuals (p.9, 11–12) and states that propensity-score methods were considered but not applied because of the limited sample size (Limitations, p.17). No additional formal sensitivity analyses (e.g., alternative adjustment sets, complete-case vs. imputed) are described — consider adding if performed.
**Results**				
Participants	**13 (a)***	Report numbers of individuals at each stage of study—eg numbers potentially eligible, examined for eligibility, confirmed eligible, included in the study, completing follow-up, and analysed	9; Fig. 1 (p.23)	"A total of 454 patients were screened for eligibility. After exclusion of patients with incomplete medical records (n=78), absence of first-line anti-VEGF–based therapy (n=139), unavailable response assessment (n=56), and progressive disease at the first response evaluation (n=87), 94 patients...were included in the landmark cohort." Figure 1 (Study flow diagram, p.23).
	**(b)**	Give reasons for non-participation at each stage	9	Reasons for exclusion given explicitly for each stage (incomplete records, no anti-VEGF therapy, unavailable response assessment, progressive disease at first evaluation), with explanation that non-returning patients accounted for unavailable response assessments (p.9).
	**(c)**	Consider use of a flow diagram	23	"Figure 1. Study flow diagram" (referenced on p.9, legend on p.23).
Descriptive data	**14 (a)***	Give characteristics of study participants (eg demographic, clinical, social) and information on exposures and potential confounders	9–10; Table 1 (p.23–24)	Overall cohort: median age 62 years, 61.7% male; maintenance vs. surveillance group comparisons (age, sex, ECOG PS, comorbidity, tumor location, metastasis type/sites, RAS status, distant LN metastasis) reported in Table 1.
	**(b)**	Indicate number of participants with missing data for each variable of interest	5	Not separately tabulated per variable; addressed at the cohort level — complete clinicopathological, treatment, and follow-up data were an inclusion requirement, and patients with incomplete records were excluded prior to analysis (n=78 of 454 screened, p.9).
	**(c)**	Cohort study—Summarise follow-up time (eg, average and total amount)	10	"The median potential follow-up duration was 75.2 months (95% CI: 41.6–92.8), as estimated using the reverse Kaplan–Meier method."
Outcome data	**15***	Cohort study—Report numbers of outcome events or summary measures over time	10–11; Table 3 (p.27)	Median PFS 11.3 vs. 6.7 months (log-rank p=0.077); median OS 24.8 vs. 18.0 months (log-rank p=0.045); 12-/24-month OS and PFS rates by group reported in Table 3.

Section	Item No.	Recommendation	Page No.	Relevant text from manuscript
Main results	**16 (a)**	Give unadjusted estimates and, if applicable, confounder-adjusted estimates and their precision (eg, 95% confidence interval). Make clear which confounders were adjusted for and why they were included	11–12; Tables 5–6 (p.30–31)	Univariable OS HR=0.60 (p=0.047) for maintenance vs. multivariable HR=0.68 (95% CI 0.37–1.26; p=0.220), adjusted for sex, comorbidity, metastatic sites, primary tumor resection, treatment response, distant LN metastasis (rationale given on p.9); analogous univariable/multivariable results for PFS (Table 6).
	**(b)**	Report category boundaries when continuous variables were categorized	10; Table 1 (p.24)	Age categorized as <65/≥65 years and <70/≥70 years (Table 1); e.g., "The proportion of patients aged ≥70 years was also significantly greater in the maintenance group (40.9% vs. 20.0%; p=0.041)."
	**(c)**	If relevant, consider translating estimates of relative risk into absolute risk for a meaningful time period	11; Table 3 (p.27)	"The 12-month OS rate was 86.1% in the maintenance group compared to 65.4% in the active surveillance group, while the 24-month OS rates were 62.7% and 40.5%, respectively. Similarly, the 12-month PFS rate was 49.3%...and 33.1%..."
Other analyses	**17**	Report other analyses done—eg analyses of subgroups and interactions, and sensitivity analyses	11; Table 4 (p.28–29)	Exploratory subgroup analyses (RAS status, comorbidity, age, metastatic burden, synchronous/metachronous disease, distant LN metastasis) and regimen-specific comparisons (Table 4), with treatment-by-subgroup interaction p-values; none reached significance.
**Discussion**				
Key results	**18**	Summarise key results with reference to study objectives	12	Opening discussion paragraph: "This study provides real-world evidence on the association between maintenance therapy strategies and survival outcomes...maintenance therapy was associated with prolonged OS...this association was attenuated after adjustment for baseline prognostic factors..."
Limitations	**19**	Discuss limitations of the study, taking into account sources of potential bias or imprecision. Discuss both direction and magnitude of any potential bias	17–18	Retrospective/single-center design and non-randomized treatment selection (selection bias/residual confounding); small sample size and limited events (imprecision, esp. subgroups); no correction for multiple comparisons; heterogeneous maintenance regimens; propensity-score methods not applied; QoL not formally assessed; 10-year enrollment period with possible temporal practice changes.
Interpretation	**20**	Give a cautious overall interpretation of results considering objectives, limitations, multiplicity of analyses, results from similar studies, and other relevant evidence	12–16, 18–19	Discussion contextualizes findings against CAIRO3, AIO KRK 0207, SAKK 41/06, KSCC 0902, AVEX, CONcePT; Conclusion (p.18–19): "...this association was observed only in the unadjusted analyses and was not maintained after multivariable adjustment, suggesting that baseline differences may have contributed to the observed findings."
Generalisability	**21**	Discuss the generalisability (external validity) of the study results	13, 17	"...real-world mCRC populations are considerably more heterogeneous...our study reflects routine clinical practice through a retrospective landmark design including a more heterogeneous population..." (p.13), balanced against the single-center design limiting external validity (p.17).

Section	Item No.	Recommendation	Page No.	Relevant text from manuscript
**Other information**				
Funding	**22**	Give the source of funding and the role of the funders for the present study and, if applicable, for the original study on which the present article is based	19	"Funding: This research received no specific grant from any funding agency in the public, commercial, or not-for-profit sectors."

** Give information separately for exposed and unexposed groups in cohort studies.*
Note: An Explanation and Elaboration article discusses each checklist item and gives methodological background and published examples of transparent reporting. The STROBE checklist is best used in conjunction with this article (freely available on the websites of PLOS Medicine, Annals of Internal Medicine, and Epidemiology). Information on the STROBE Initiative is available at www.strobe-statement.org.
