# Supplementary material for: Association of Maintenance Therapy Strategies with Survival Following First-Line Bevacizumab-Based Combination Chemotherapy in Metastatic Colorectal Cancer: A Single-Center Retrospective Observational Study
Source: J Clin Med. 2026 Aug 13;15(16):6279. doi: 10.3390/jcm15166279 (PMC13513069; doi:10.3390/jcm15166279)
Supplement: Supplementary file 1 [file jcm-15-06279-s001.zip › Supplementary Tables S1 and S2.pdf]

Supplementary Table S1. Detailed Clinical, Pathological, and Treatment Characteristics

Variable	Anti-VEGF Maintenance (+) (n=44)	Anti-VEGF Maintenance (−) (n=50)	p değeri
**FAMILY HISTORY**			
**Family History**			
Unremarkable	16 (37.2%)	20 (40.8%)	0.919 $^{X^2}$
Family history of CRC	5 (11.6%)	4 (8.2%)	
Family history of non-CRC cancer	9 (20.9%)	13 (26.5%)	
Unknown	12 (27.9%)	11 (22.4%)	
**TUMOR AND PATHOLOGICAL CHARACTERISTICS**			
**Detailed Tumor Location**			
Cecum	6 (13.6%)	6 (12.0%)	
Ascending colon	6 (13.6%)	3 (6.0%)	
Transverse colon	2 (4.5%)	4 (8.0%)	
Descending colon	4 (9.1%)	2 (4.0%)	
Sigmoid colon	8 (18.2%)	7 (14.0%)	
Rectum	9 (20.5%)	19 (38.0%)	
Rectosigmoid	9 (20.5%)	9 (18.0%)	
**Histological Type**			
Adenocarcinoma NOS	40 (90.9%)	43 (86.0%)	0.434 $^{X^2}$
Special Histological Subtype	4 (9.1%)	7 (14.0%)	
Mucinous	2	6	
Signet-ring cell	2	1	
**Baseline CEA Level (ng/mL)**			
Median (IQR)	14.9 (2.6-108.5)	6.0 (3.3-41.9)	0.921 ^{m}
Min - Max	0.80 - 3090	1.00 - 6680	
**Baseline CA 19-9 Level (U/mL)**			
Median (IQR)	33.9 (11.9-102.2)	18.9 (6.8-180.9)	0.499 ^{m}
Min - Max	0.60 - 8342	0.60 - 10000	
**Clinical Stage (TNM)**			0.963 X^2
Stage 2A	1 (2.3%)	2 (4.0%)	
Stage 3B	5 (11.4%)	4 (8.0%)	
Stage 3C	1 (2.3%)	2 (4.0%)	
Stage 4A	19 (43.2%)	20 (40.0%)	
Stage 4B	11 (25.0%)	12 (24.0%)	
Stage 4C	7 (15.9%)	10 (20.0%)	
**METASTATIC CHARACTERISTICS**			
**Time from Diagnosis to Metastasis (Months)**			
Mean ± SD	3.4 ± 10.2	2.4 ± 6.5	0.786 ^{m}
Median (Min-Max)	0 (0-60)	0 (0-31)	

**Metastatic Sites**			
Liver	31 (70.5%)	41 (82.0%)	0.226 $^{\chi^2}$
Lung	18 (40.9%)	12 (24.0%)	0.120 $^{\chi^2}$
Peritoneum	12 (27.3%)	12 (24.0%)	0.814 $^{\chi^2}$
Bone	1 (2.3%)	5 (10.0%)	0.209 $^{\chi^2}$
Brain	0 (0.0%)	1 (2.0%)	1.000 $^{\chi^2}$
Distant lymph node	3 (6.8%)	13 (26.0%)	**0.015** $^{\chi^2}$
Kidney	1 (2.3%)	0 (0.0%)	0.468 $^{\chi^2}$
Adrenal	1 (2.3%)	0 (0.0%)	0.468 $^{\chi^2}$
**Number of Liver Metastases**			
No	13 (29.5%)	10 (20.0%)	
1	10 (22.7%)	13 (26.0%)	
2	6 (13.6%)	9 (18.0%)	
3	0 (0.0%)	3 (6.0%)	
4	0 (0.0%)	3 (6.0%)	
5	1 (2.3%)	1 (2.0%)	
6	1 (2.3%)	0 (0.0%)	
Multiple	13 (29.5%)	11 (22.0%)	
**SECOND MALIGNANCY AND SURVIVAL STATUS**			
Second Primary Malignancy	3 (6.8%)	6 (12.0%)	
**NEOADJUVANT / ADJUVANT TREATMENT**			
Received Neoadjuvant CT	1 (2.3%)	2 (4.0%)	
Received Adjuvant CT	10 (22.7%)	8 (16.0%)	
Received Radiotherapy	5 (11.4%)	6 (12.0%)	
**METASTATIC SECOND-LINE TREATMENT**			
Received Second-Line Treatment	23 (52.3%)	22 (44.0%)	
**2L Chemotherapy Regimen**	**n=23**	**n=22**	
CAPOX	2 (8.7%)	2 (9.1%)	
CAPIRI	4 (17.4%)	4 (18.2%)	
FOLFOX	5 (21.7%)	2 (9.1%)	
FOLFİRİ	11 (47.8%)	12 (54.5%)	
FOLFOXİRİ	1 (4.3%)	2 (9.1%)	
**2L Specific Biologic Agent**			
Not received	3 (13.0%)	1 (4.5%)	
Bevacizumab	15 (65.2%)	17 (77.3%)	
Panitumumab	1 (4.3%)	1 (4.5%)	
Setuksimab	2 (8.7%)	2 (9.1%)	
Aflibercept	2 (8.7%)	1 (4.5%)	
**2L Number of Cycles**			
Mean ± SD	8.6 ± 4.8	6.8 ± 4.2	
Median (Min-Max)	6 (2-19)	6 (1-18)	

**2L Treatment Response**	**n=23**	**n=22**	
Complete Response (CR)	2 (8.7%)	3 (13.6%)	
Partial Response (PR)	0 (0.0%)	2 (9.1%)	
Stable Disease (SD)	0 (0.0%)	1 (4.5%)	
Progressive Disease (PD)	19 (82.6%)	10 (45.5%)	
**METASTATIC THIRD-LINE TREATMENT**			
Received Third-Line Treatment	16 (36.4%)	10 (20.0%)	-
**3L Chemotherapy Regimen**	n=16	n=10	
CAPOX	1 (6.2%)	0 (0.0%)	
CAPIRI	1 (6.2%)	0 (0.0%)	
FOLFOX	5 (31.2%)	3 (30.0%)	
FOLFİRİ	4 (25.0%)	2 (20.0%)	
Regorafenib	4 (25.0%)	5 (50.0%)	
FOLFOXİRİ	1 (6.2%)	0 (0.0%)	
**3L Biologic Agent**	n=16	n=10	
Not received	7 (43.8%)	5 (50.0%)	
Bevacizumab	6 (37.5%)	3 (30.0%)	
Panitumumab	0 (0.0%)	1 (10.0%)	
Setuksimab	2 (12.5%)	1 (10.0%)	
Aflibercept	1 (6.2%)	0 (0.0%)	
**3L Number of Cycles**			
Mean ± SD	5.6 ± 3.7	4.7 ± 3.6	
Median (Min-Max)	5 (1-12)	4 (1-12)	

^{m} Independent samples Mann-Whitney U test, $^{X^2}$ Chi-square test / Fisher exact test, $p < 0.05$ was considered statistically significant.

Supplementary Table S2. Treatment-Related Adverse Events During First-Line Therapy According to Subsequent Treatment Strategy

Variable	Anti-VEGF Maintenance (+) (n=44)	Anti-VEGF Maintenance (–) (n=50)	p-value
**Any adverse event**	31 (70.5%)	18 (36.0%)	**0.001** $^{X^2}$
**CHEMOTHERAPY-RELATED ADVERSE EVENTS**			
Peripheral neuropathy	14 (31.8%)	8 (16.0%)	0.089 $^{X^2}$
Neutropenia	12 (27.3%)	5 (10.0%)	**0.035** $^{X^2}$
Thrombocytopenia	14 (31.8%)	9 (18.0%)	0.151 $^{X^2}$
Diarrhea	3 (6.8%)	1 (2.0%)	0.337 $^{X^2}$
Constipation	0 (0.0%)	1 (2.0%)	1.000 $^{X^2}$

Mucositis	1 (2.3%)	0 (0.0%)	0.468 $^{X^2}$
Infusion reaction	1 (2.3%)	0 (0.0%)	0.468 $^{X^2}$
Intolerance	1 (2.3%)	0 (0.0%)	0.468 $^{X^2}$
**Maximum chemotherapy toxicity grade**			
Grade 0	14 (31.8%)	30 (60.0%)	**<0.001** ^{m}
Grade 1	3 (6.8%)	10 (20.0%)	
Grade 2	21 (47.7%)	7 (14.0%)	
Grade 3	6 (13.6%)	3 (6.0%)	
**Chemotherapy treatment modification**			
None	27 (61.4%)	41 (82.0%)	
Dose reduction	9 (20.5%)	1 (2.0%)	
Treatment discontinuation	5 (11.4%)	3 (6.0%)	
Treatment break	2 (4.5%)	3 (6.0%)	
Dose reduction + break	1 (2.3%)	1 (2.0%)	
Any treatment modification	17 (38.6%)	8 (16.0%)	**0.019** $^{X^2}$
**BEVACIZUMAB-RELATED ADVERSE EVENTS**			
Hypertension	2 (4.5%)	0 (0.0%)	0.216 $^{X^2}$
Proteinuria	1 (2.3%)	0 (0.0%)	0.468 $^{X^2}$
Thromboembolic event	1 (2.3%)	0 (0.0%)	0.468 $^{X^2}$
Perforation	0 (0.0%)	1 (2.0%)	1.000 $^{X^2}$
Skin rash	0 (0.0%)	1 (2.0%)	1.000 $^{X^2}$
Diarrhea	3 (6.8%)	0 (0.0%)	0.099 $^{X^2}$
**Any bevacizumab-related AE**	6 (13.6%)	3 (6.0%)	0.297 $^{X^2}$
**Maximum bevacizumab toxicity grade**			0.202 ^{m}
Grade 0	38 (86.4%)	46 (92.0%)	
Grade 1	3 (6.8%)	3 (6.0%)	
Grade 2	1 (2.3%)	0 (0.0%)	
Grade 3	2 (4.5%)	0 (0.0%)	
**Bevacizumab treatment modification**			0.246 $^{X^2}$
None	39 (88.6%)	48 (96.0%)	0.246 $^{X^2}$
Treatment discontinuation	4 (9.1%)	2 (4.0%)	0.414 $^{X^2}$
Treatment break	1 (2.3%)	0 (0.0%)	0.468 $^{X^2}$

$^{X^2}$ Chi-square test / Fisher exact test, ^{m} Mann-Whitney U test, $p < 0.05$ was considered statistically significant
